# Prediction of Methylene Blue Removal by Nano TiO_2_ Using Deep Neural Network

**DOI:** 10.3390/polym13183104

**Published:** 2021-09-15

**Authors:** Nesrine Amor, Muhammad Tayyab Noman, Michal Petru

**Affiliations:** Department of Machinery Construction, Institute for Nanomaterials, Advanced Technologies and Innovation (CXI), Technical University of Liberec, Studentská 1402/2, 461 17 Liberec 1, Czech Republic; michal.petru@tul.cz

**Keywords:** artificial neural network, titanium dioxide nanoparticles, methylene blue dye removal

## Abstract

This paper deals with the prediction of methylene blue (MB) dye removal under the influence of titanium dioxide nanoparticles (TiO${}_{2}$ NPs) through deep neural network (DNN). In the first step, TiO${}_{2}$ NPs were prepared and their morphological properties were analysed by scanning electron microscopy. Later, the influence of as synthesized TiO${}_{2}$ NPs was tested against MB dye removal and in the final step, DNN was used for the prediction. DNN is an efficient machine learning tools and widely used model for the prediction of highly complex problems. However, it has never been used for the prediction of MB dye removal. Therefore, this paper investigates the prediction accuracy of MB dye removal under the influence of TiO${}_{2}$ NPs using DNN. Furthermore, the proposed DNN model was used to map out the complex input-output conditions for the prediction of optimal results. The amount of chemicals, i.e., amount of TiO${}_{2}$ NPs, amount of ehylene glycol and reaction time were chosen as input variables and MB dye removal percentage was evaluated as a response. DNN model provides significantly high performance accuracy for the prediction of MB dye removal and can be used as a powerful tool for the prediction of other functional properties of nanocomposites.

## 1. Introduction

Modern world witnesses the miracles of nanotechnology as it manipulates matter on molecular level with at least having one dimension less than 100 nm [1,2]. Researchers are applying nanomaterials i.e., nanoparticles, nanowires, nanorods, nanosheets, nanoflowers etc in medicines, optical instruments, energy devices, civil and building industry, aeronautics and electronics for better performance [3,4,5,6]. TiO${}_{2}$ is a functional material mostly used as a photo catalyst in industrial applications [7,8,9]. TiO${}_{2}$ nanomaterials have been investigated for photodegradation of organic pollutants i.e., tetracycline and MB [10,11], self-cleaning coatings, antimicrobial coatings, sensors and for other purposes by the academic researchers and industrial experts [12,13,14]. In an experimental study, Noman et al. worked with the synthesis and single step coating of TiO${}_{2}$ NPs on cotton to develop photocatalytically active cotton composites for antimicrobial and self-cleaning applications. They used sonication method and reported the average particle size for their samples 4 nm [15]. On the other hand, DNN models have achieved human-level performance and have shown great success in different real-world applications, including computer vision [16], textile process, biomedical engineering [17], material engineering [18]. DNN is an efficient machine learning tool suitable for the prediction of output parameters from input variables where there is an unknown relationship exists between input and output variables [19,20,21]. In recent years, DNN has been widely used to predict various properties of textiles. Lui et al. developed a new strategy to predict the initial failure strength criterion of woven fabric reinforced composites based on micro-mechanical model by modifying DNN and mechanics of structure genome (MSG) [22]. MGS is used to perform initial failure analysis of a square pack microscale model that trained the samples to detect yarn failure criterion. The effectiveness of this strategy was confirmed by testing yarns of mesoscale plain weave fabrics and fiber reinforced composite materials to compute the initial failure strength constants. Khude et al. applied artificial neural network (ANN) and adaptive network-based fuzzy inference system (ANFIS) to predict antimicrobial properties of knitted fabrics made with silver fibres [23]. Both studies reported good results during training and testing of datasets. However, ANFIS showed better performance with small datasets. Altarazi et al. used multiple algorithms i.e., stochastic gradient descent (SGD), ANN, k-nearest neighbors (kNN), decision tree (DT), regression analysis, support vector machine (SVM), random forest (RF), logistic regression (LoR) and AdaBoost (AB), for the prediction and classification of tensile strength of polymeric films with various compositions [24]. The obtained results show that SVM algorithm has superior predictive ability. In addition, the experimental results show that classification ability of used algorithms was excellent for sorting films into conforming and non-conforming parts. Yang et al. identified knitted fabric pilling behavior by modifying ANN into deep principle components analysis-based neural networks (DPCANN) [25]. In DPCANN, principal components automatically track down the fabric properties before and after pilling test and then neural network was applied to evaluate pilling grades. The obtained results elucidate that DPCANN has above average classification efficiency for pilling behavior of knitted fabric.

Many other researchers worked with DNN in textiles. Li et al. proposed Fisher criterion-based deep learning algorithm for defects detection of patterned fabrics [26]. A Fisher criterion-based stacked denoising method has been used for fabric images to classify into defective and defectless categories. The experimental results showed that the accuracy of proposed approach in defects detection is excellent for patterned fabrics and more complex jacquard warp-knitted fabric. Ni et al. proposed a novel online algorithm that detects and predicts the coating thickness on textiles by hyperspectral images [27]. The proposed algorithm was based on two different optimization modules i.e., the first module is called extreme learning machine (ELM) classifier whereas, the later is called a group of stacked autoencoders.The ELM module optimized by a new optimizer known as grey wolf optimizer (GWO), to determine the number of neurons and weights to get more accuracy while detection and classification. The results explained that online detection performance significantly improved with a combination of the variable-weighted stacked autoencoders (VW-SAE) with GWO-ELM that provide 95.58% efficiency. Lazzús et al. used the combined ANN with particle swarm optimization (PSO) to predict the thermal properties [28]. The results demonstrated that the proposed model ANN-PSO provided better results than feedforward ANN. Malik et al. applied ANN to predict yarns crimp for woven fabrics. Simulation results showed a good prediction accuracy, especially for warp yarn [29]. Lu et al. used ANN and multiple linear regression (MLR) based on acoustic emission detection for the prediction of tensile strength of single wool fibers [30]. The coefficients of determination of ANN and MLR show that there is a high correlation between the predicted and measured values of strength of wool. However, ANN model has the higher accuracy and lower error prediction compared to MLR. In another study, Tadesse et al. proposed ANN and fuzzy logic (FL) to predict the tactile comfort of functional fabrics parameters [31]. FL has been performed to predict the predicted hand values (HVs) from finishing parameters; then, the total hand values (THVs) has been predicted from the HV using FL and ANN model. FL provided an efficient prediction of the HV with lower relative mean percentage error (RMPE) and root mean square error (RMSE). In addition, the fuzzy logic model (FLM) and ANN models showed an effective prediction performance of the THV with lower RMSE and RMPE values. Mishra used ANN models during the production of cotton fabric for the prediction of yarn strength utilization [32]. The selected input parameters were yarn counts, initial crimps, total number of yarns and yarn strengths in both longitudinal and transverse directions along with the weave float length. The experimental results showed that yarn strength utilization percentage increased with an increase in yarn number in both directions, however, a decrease in crimp percentage and float length was observed. El-Geiheini et al. worked with different types of yarns and used ANN and image processing tools for modeling and simulation of yarn tenacity and elongation [33]. They reported that the proposed techniques are suitable for the estimation of various yarn properties with minimum error. Erbil et al. used ANN and regression tools for tensile strength prediction of ternary blended open-end rotor yarns [34]. They performed stepwise multiple linear regression (MLR) method and trained their ANN algorithm with Levenberg–Marquardt backpropagation function. Furthermore, they performed a comparison of both models for prediction efficiency. The reported results of their experimental study demonstrated that ANN models give a better prediction output than MLR for both parameters i.e., breaking strength and elongation at break. Lui et al. developed a new strategy to predict the initial failure strength criterion of woven fabric reinforced composites based on micromechanical model by modifying deep learning neural network (DNN) and mechanics of structure genome (MSG) [22]. MGS is used to perform initial failure analysis of a square pack microscale model that trained the samples to detect yarn failure criterion. The effectiveness of this strategy was confirmed by testing yarns of mesoscale plain weave fabrics and fiber reinforced composite materials to compute the initial failure strength constants. The literature shows that the prediction of mechanical behavior of composites materials is a complex task due to variations in boundary conditions and structures [5,35]. Wang et al. presented the prediction of the tensile strength of ultrafine glass fiber felts by ANN [36]. The tensile strength are modelled based on the mean diameter of fibers, bulk density and resin content. Simulation results showed that ANN model provided a high prediction accuracy and lower mean relative errors. Unal et al. selected single jersey knitted fabrics for the evaluation of air permeability and combined ANN algorithm with regression methods for the prediction of bursting strength of used knit structures [37]. Implementation of results showed that both methods were able to predict precisely the properties of knitted fabrics. However, ANN had a slightly positive edge when used for prediction. Recently, Amor et al. used ANN and MLR for the prediction of functional properties of nano TiO${}_{2}$ coated cotton composites and their results show that ANN outperformed MLR for prediction accuracy [38].

The discussed literature reveal that ANN is mostly used machine learning tool for textile industry [29,34,38,39]. DNN is a category of ANN model with multiple layers between input and output layers. DNN takes an edge of automatic learning process. Therefore, main contributions of this paper are to investigate the accuracy of DNN model for the prediction of MB dye removal under the influence of TiO${}_{2}$ NPs and compare the results with MLR.

This study is organized as follows: Section 2 describes materials, synthesis of TiO${}_{2}$ NPs and explanation of DNN model framework. Section 3 discusses the simulation, comparison and results for the prediction of MB dye removal. Section 4 summarizes the main findings and concludes the paper.

## 2. Materials and Methods

### 2.1. Materials

All chemicals and reagents for the synthesis of TiO${}_{2}$ NPs i.e., titanium tetraisopropoxide (TTIP), ethylene glycol (EG) and for photodegradation study i.e., MB dye were received from Sigma-Aldrich (Prague, Czech Republic) and used without any further purification. The experimental design with different amount of TTIP and EG is presented in Table 1.

### 2.2. Synthesis of TiO${}_{2}$ NPs

**Figure 1:**
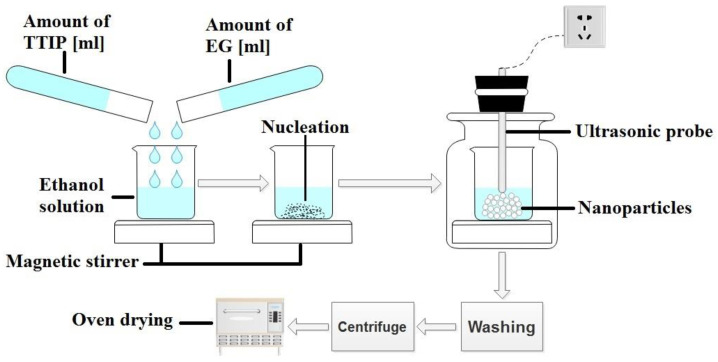
Graphical representation of experimental setup for the synthesis of TiO${}_{2}$ NPs.

Initially, TTIP and EG were added in a beaker containing 50 mL ethanol. The molar ratio of TTIP: EG was 2:1. The mixture was magnetically stirred at 500 rpm for 15 min. The mixture was then sonicated by ultrasonic probe (Bandelin Sonopuls HD 3200, 20 kHZ, 200 W, Berlin, Germany) for time intervals based on the experimental design. The temperature was maintained at 80 ${}^{\circ }$C by using hot plate equipped with magnetic stirrer. The resulting nanoparticles were washed with ethanol to remove impurities and then centrifuged at 4000 rpm for 5 min to remove liquid from inside. The resulting nanoparticles were further dried in an oven at 100 ${}^{\circ }$C for 2 h. The experimental setup is shown in Figure 1.

### 2.3. MB Removal

The photocatalytic activity of TiO${}_{2}$ NPs was evaluated by the discoloration of MB solution under UV light. In this experiment, 1 g L${}^{-1}$ TiO${}_{2}$ NPs were mixed in 50 mL solution containing 100 mg L${}^{-1}$ MB dye. The solution was stirred by a magnetic stirrer and placed in the dark for 40 min to reach an adsorption desorption equilibrium. The UV light source was a 500 W UV lamp with light intensity 30 W m${}^{-2}$. MB residual concentration was calculated by spectrophotometer at 668 nm wavelength. The color removal efficiency (*CR*%) was evaluated by the given Equation (1):

(1) $$CR\%=\left[1-\frac{C}{C_{0}}\right]*100,$$

where $C_{0}$ and *C* represents the initial and final concentration of MB in the solution respectively. The initial spectrum of dye solution without TiO${}_{2}$ NPs was taken as standard sample. An aliquot was taken out by a syringe after a fix time interval to evaluate the results of MB removal.

### 2.4. Deep Neural Network

ANN model is widely used in the prediction of functional properties of composite structures [38,40]. Generally, ANN model contains three layers and ANN model with more than hidden layers is known as DNN model [18]. DNN is widely used to investigate the correlation between variables for critical problems [41]. Automatic creation and exploration of information from previous learning is an interesting feature of DNN [42]. DNN tunes the weights constantly until predicted and target values match. Back-propagation calculates the error between predicted and targeted and update weights to remove error after iterations [18,22]. The equation of DNN is given below:

(2) $$y=f\left(\sum_{i}W_{ij}*X_{i}+b_{j}\right),$$

where, *y* represents the output. $X_{i}$ represents the *i*th input variables. $W_{ij}$ represents the weight and $b_{j}$ is the bias. The weights and biases include the information that neuron recovers during training. φ is the activation function mostly employed sigmoid activation function given in Equation (3) [43]:

(3) $$f\left(x\right)=\frac{1}{1+e^{\delta x}},$$

where δ denotes the sigmoid function steepness parameter and *x* is given by

(4) $$x=\sum_{i}W_{i}*X_{i},$$

**Figure 2:**
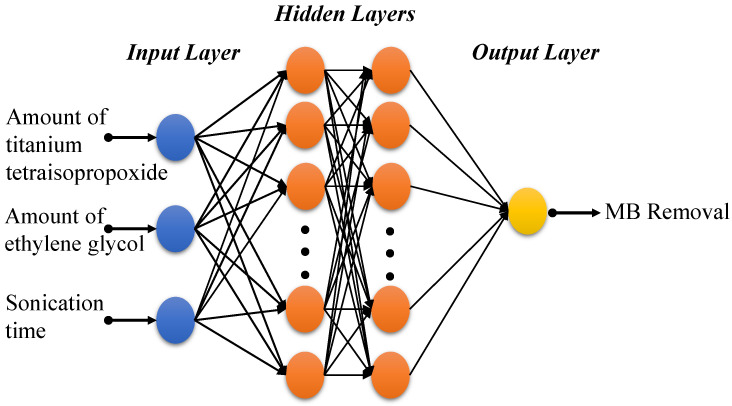
DNN model for the prediction of of the removal of methylene blue dye under the influence of nano TiO${}_{2}$.

A detail tutorial of DNN algorithms are presented by various researchers in their studies [44,45]. In the present work, the DNN model has been developed by a multi-layer feed-forward network. The hyperbolic tangent sigmoid function is used as the activation function for each layer. The amount of titanium precursor, amount of ehylene glycol and process time are taken as the input variables whereas the MB removal are outputs variable as described in Figure 2.

The DNN model has been trained by the Bayesian regularization backpropagation algorithm. 85% of the data was used for the training of the DNN model and 15% of the data was used for testing. After several tentative simulation, it was found that the best DNN architecture that gives the lowest relative error and highest correlation coefficient has a structure of 3-12-12-6-1, which represents three variables in the input layer, three hidden layers with 12, 12 and 6 neurons respectively, and one predicted output.

### 2.5. Evaluation of DNN Model

The performance and accuracy of the DNN model was evaluated using different method including mean absolute error (*MAE*), mean squared error (*MSE*), root mean squared error (*RMSE*), standard deviation (*SD*) of the error and coefficient of correlation ($R^{2}$), and they are expressed respectively as follows:

(5) $$MAE=\frac{1}{n}\sum_{i=1}^{n}\left|\left(y_{i}-{\hat{y}}_{i}\right)\right|,$$

(6) $$MSE=\frac{1}{n}\sum_{i=1}^{n}{\left(y_{i}-{\hat{y}}_{i}\right)}^{2},$$

(7) $$RMSE=\sqrt{\frac{1}{n}\sum_{i=1}^{n}{\left(y_{i}-{\hat{y}}_{i}\right)}^{2}},$$

(8) $$SD=\sqrt{\frac{1}{n-1}\sum_{i=1}^{n}{\left({\left(y-\hat{y}\right)}_{i}-\bar{\left(y-\hat{y}\right)}\right)}^{2}},$$

(9) $$R^{2}={\left(\frac{\sum_{i=1}^{n}\left(y_{i}-\bar{y}\right)\left(\hat{y_{i}}-\bar{\hat{y}}\right)}{\sqrt{\sum_{i=1}^{n}{\left(y_{i}-\bar{y}\right)}^{2}}\sqrt{\sum_{i=1}^{n}{\left(\hat{y_{i}}-\bar{\hat{y}}\right)}^{2}}}\right)}^{2}.$$

where $y_{i}$ and $\hat{y}$ are the actual and predicted outputs, respectively. $\bar{y}$ is the mean of the actual variables and $\bar{\hat{y}}$ represents the mean of the predicted variables. *n* is the number of samples.

In addition, statistical analysis (ANOVA) has been performed to test the statistical significance of input and output variables [38,46,47].

## 3. Results and Discussion

### 3.1. Scanning Electron Microscopy (SEM) Analysis

**Figure 3:**
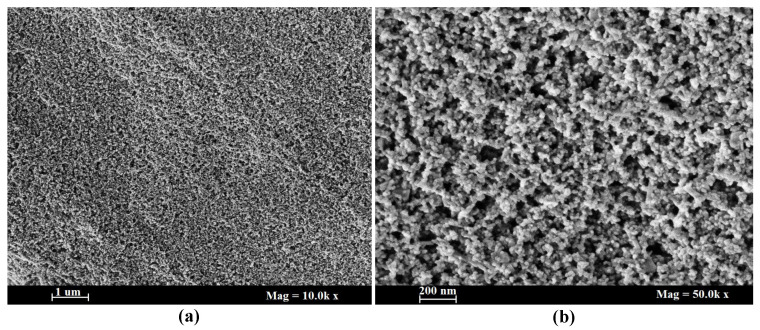
SEM images of as synthesized TiO${}_{2}$ NPs (**a**) at magnification 10.0 k × and (**b**) at magnification 50.0 k ×.

The results of SEM analysis for the synthesis of TiO${}_{2}$ NPs are presented in Figure 3. It is observed from SEM results that the synthesized TiO${}_{2}$ NPs are quasi spherical in shape and homogeneously distributed. The estimated size of the particles by image analysis was 20 nm. Moreover, for texture properties, the randomly selected samples were examined by Brunauer-Emmett Teller (BET surface area and pore size analyzer Quantachrome—NOVA 2200e (Boynton Beach, FL, USA)), Atomic Force Microscopy (AFM) system (Park System${}^{\mathrm{TM}}$, Suwon, South Korea) and Dynamic Light Scattering (DLS Malvern Pananalytical Zetasizer Ultra (Malvern, UK)) techniques and the obtained results are presented in Table 2.

In addition, XRD analysis was carried out to confirm the crystallite size and the purity of the crystalline phase. XRD results are illustrated and discussed (see the Supporting Information).

### 3.2. Evaluation of MB Removal

MB discoloration was investigated with 1 g L${}^{-1}$ TiO${}_{2}$ NPs and with 100 mg L${}^{-1}$ initial concentration of MB. The results of MB removal explain that a complete discoloration of MB was done within 40 min under UV light. In order to confirm that this change was due to the presence of TiO${}_{2}$ NPs and not by the poor light fastness of MB, dye solution was exposed to UV light without TiO${}_{2}$ NPs. This solution didn’t change its color even for longer irradiations time. Therefore, it is confirmed that TiO${}_{2}$ NPs are highly photo active as their minimal quantity discolor MB solution in a short span of time.

### 3.3. Analysis of the Proposed DNN Model

We applied the DNN to predict the removal of methylene blue dye under the influence of titanium dioxide. After many trials, the best results that include the lower MSE and high prediction performance of the methylene blue removal obtained from a DNN model with five-layers, i.e., an input, three hidden, and an output layers, and the number of hidden layer nodes are 12, 12 and 6, where the network provides highly accurate results. The transfer functions used for hidden and output layers in this work is the type of tangent sigmoid (tansig) function. The best selection of a transfer function for input and output layers guarantee the accuracy of the predicted results. In addition, Bayesian regularization backpropagation was used to train the DNN. The setting of training parameters of the DNN are presented in Table 3. The obtained results with the DNN model were compared with MLR.

**Figure 4:**
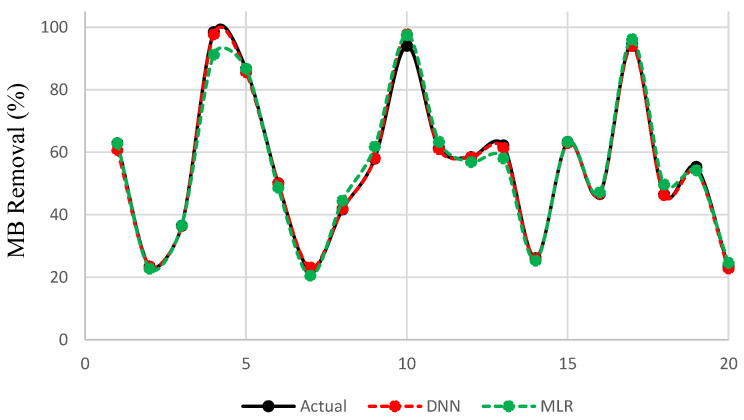
The predicted and actual values using DNN and MLR.

**Figure 5:**
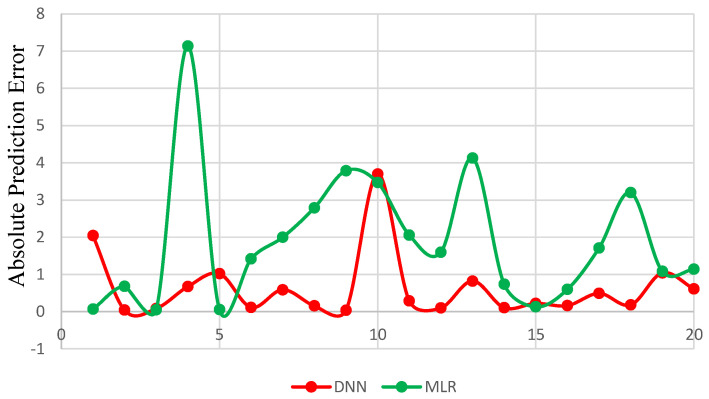
Results of absolute error for actual and predicted values using DNN and MLR models.

Figure 4 illustrates the predicted values of MB dye removal using DNN and MLR. Figure 5 shows the absolute prediction error given by the difference between predicted and actual values using both MLR and DNN. We noticed that the values of prediction error were significantly lower for the DNN model as compared to MLR. It is clear from the Figure 5 that MLR model has one error burst at sample number 4 and has higher errors for the prediction of most values. Table 4 represents the computed MSE, RMSE, MAE, SD and $R^{2}$ for both used models to predict the methylene blue removal. We observed that the proposed DNN model outperformed MLR with lower errors according to MSE, RMSE, MAE, SD, and high accuracy according to $R^{2}$.

**Figure 6:**
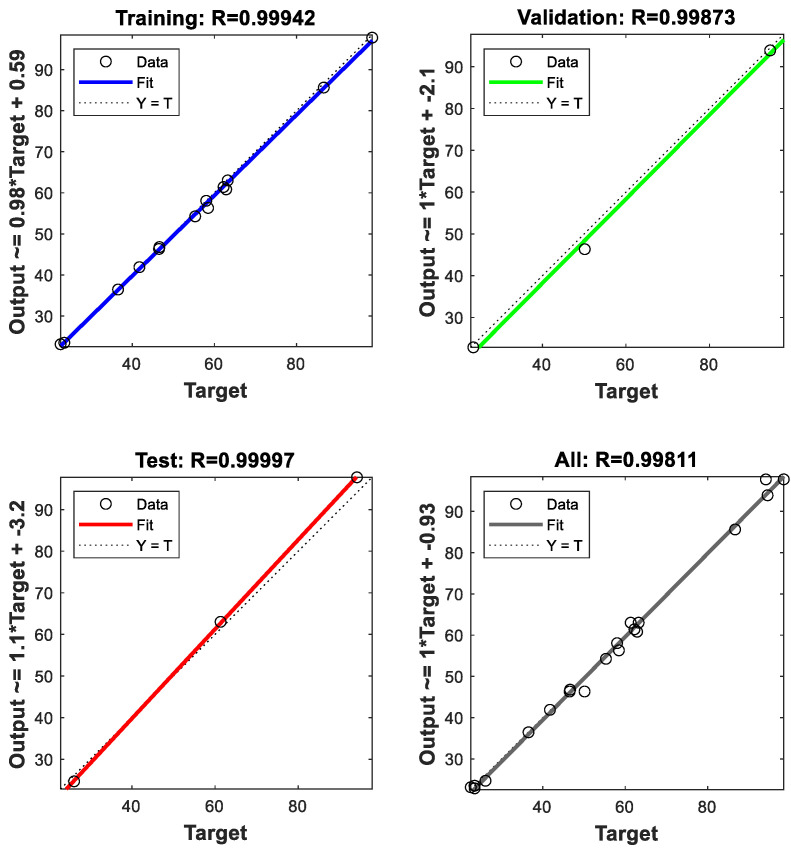
Correlation coefficient for experimental and predicted values by back-propagation DNN model.

**Figure 7:**
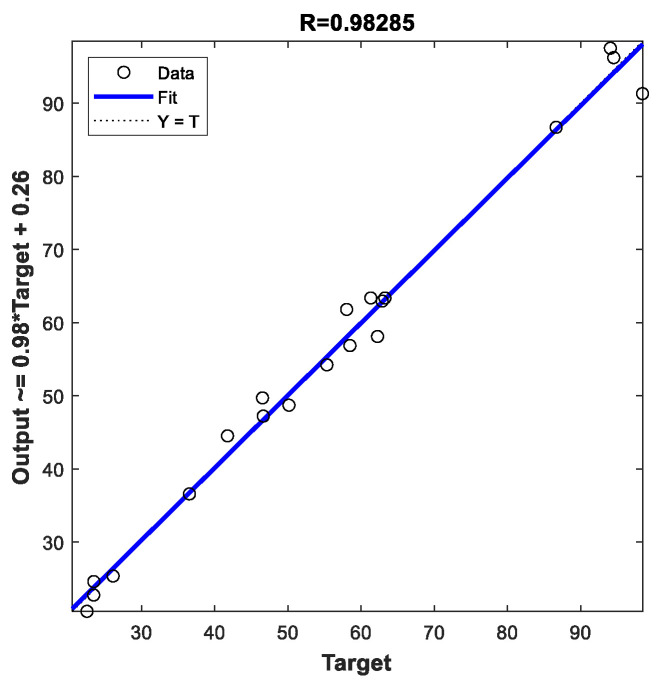
Correlation of actual and predicted values for all data sets by MLR model.

The correlation between actual and predicted values using DNN for training and validation of all data sets is illustrated in Figure 6. It is observed from the results that the correlation coefficients (*R*-value) provide an excellent correlation between the predicted and actual values, where R>99% which confirm the highly prediction accuracy of DNN. Figure 7 showed the correlation between the actual and predicted values using MLR model. We noticed that MLR model provide a good prediction accuracy for the MB removal, where R=98%. It is clear from the obtained results that both DNN and models verifies their accuracy and effectiveness in the prediction process. However, the performance of DNN model outperformed MLR with higher accuracy and lower errors.

One-way ANOVA was used to check the robustness of the results obtained through DNN, MLR and experiments. ANOVA analysis helps to understand the relationship between predicted values of MB dye removal under the influence of nano TiO${}_{2}$ with all process variables. Table 5 shows ANOVA results for MB dye removal obtained by DNN, MLR and experiments. The overall results show that DNN model is statistically more significant than experimental values and MLR values as DNN provides lowest *p*-value.

## 4. Conclusions

In this paper, we introduced DNN model for the prediction of MB dye removal under the influence of TiO${}_{2}$ NPs. In the fist phase, TiO${}_{2}$ NPs were successfully synthesized by sonication. Scanning electron microscopy results showed quasi spherical shape of all prepared samples. The particles were homogeneous and successfully used in MB dye removal. The prediction of MB dye removal was performed by DNN model. In comparison, DNN showed more accurate results than MLR model. The obtained results for MSE, RMSE, MAE and SD elucidate that DNN model has lower error than MLR. The successful utilization of DNN model shows a non-linear behaviour for the prediction of MB dye removal. The obtained results confirm that DNN model can also be effectively used for the prediction of MB dye removal and for the removal of other organic pollutants in different industries.

## Figures and Tables

**Table 1:** Experimental design and variables for the synthesis of TiO${}_{2}$ NPs.

Sample	Amount of TTIP [mL]	Amount of EG [mL]	Sonication Time [h]
1	10	8	4
2	1	2	0.5
3	1	2	4
4	10	4	1
5	10	5	0.5
6	5	5	2
7	1	8	4
8	5	5	0.5
9	7	5	1
10	10	2	0.5
11	7	5	2
12	7	8	1
13	5	2	2
14	1	4	1
15	7	5	2
16	5	5	1
17	10	2	4
18	5	5	2
19	7	5	4
20	1	8	0.5

**Table 2:** Textural and microstructural properties of TiO${}_{2}$ NPs.

Sample	Surface Area [m${}^{2}$/g]	Pore Volume [cm^3^/g]	Surface Roughness [nm]	Hydrodynamic Diameter [nm]
1	187±4	0.17±0.5	5.24±2	28±3
6	163±2	0.21±1	2.11±1	31±1
9	149±5	0.26±0.5	1.38±1	24±2
16	201±4	0.13±0.5	3.81±2	41±1

**Table 3:** Parameters and settings of training network.

Parameters	Settings
Training function	trainbr
Transfer function of hidden layers	tansig, tansig, tansig
Transfer function of output layer	tansig
Epochs	1000
Input node	3
Hidden node	12,12, 6
Output node	1
Performance goal	0.00001

**Table 4:** The performance measures of DNN and MLR.

Methods	*MSE*	*RMSE*	*MAE*	*SD*	$R^{2}$
DNN (training)	1.1186	1.0576	0.6254	1.1719	0.9997
DNN (testing)	1.09958	1.0532	0.6213	0.3558	0.9999
MLR	6.6044	2.5699	1.8933	2.6366	0.9882

**Table 5:** Analysis report of DNN, MLR and experimental values for methylene blue removal.

Methods	*p*-Value	F-Value
DNN	$1.73\times 10^{-10}$	97.31
MLR	$2.821\times 10^{-9}$	66.97
Experimental	$1.771\times 10^{-9}$	71.33

## Data Availability

Not applicable.

## Supplementary Materials

The following are available online at https://www.mdpi.com/article/10.3390/polym13183104/s1, Figure S1: XRD patterns of randomly selected samples i.e., Sample 1, Sample 6, Sample 9 and Sample 16 of TiO${}_{2}$ NPs.
