# Supplementary material for: Prediction of Methylene Blue Removal by Nano TiO2 Using Deep Neural Network
Source: Polymers (Basel). 2021 Sep 15;13(18):3104. doi: 10.3390/polym13183104 (PMC8473325; doi:10.3390/polym13183104)
Supplement: Supplementary file 1 [file polymers-13-03104-s001.zip › polymers-1379513-supplementary.pdf]

Prediction of Methylene Blue Removal by Nano TiO_2 Using Deep Neural Network

Nesrine Amor[a*], Muhammad Tayyab Noman[a*], Michal Petru[a]

[a] Department of Machinery Construction, Institute for Nanomaterials, Advanced Technologies and Innovation (CXI), Studentská 1402/2, 461 17 Liberec 1, Technical University of Liberec, Czech Republic.

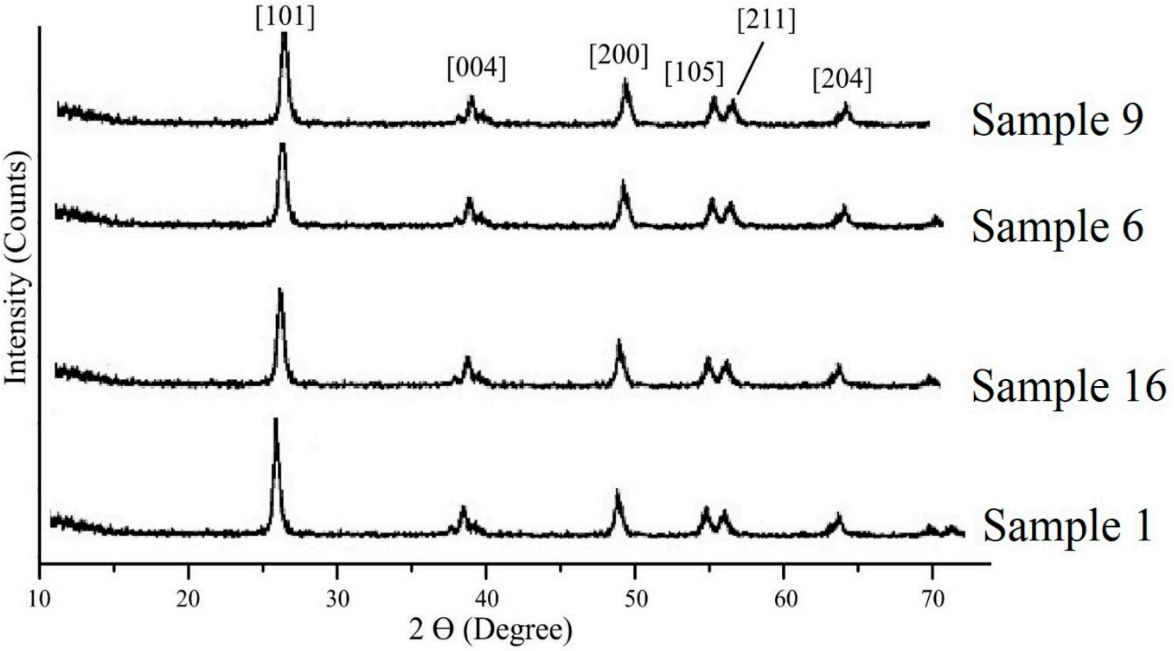

Figure S1. XRD patterns of randomly selected samples i.e., Sample 1, Sample 6, Sample 9 and Sample 16 of TiO_2 NPs.

XRD patterns for all randomly selected samples (Sample 1, Sample 6, Sample 9 and Sample 16) confirmed the existence of pure anatase crystals of nano TiO_2. The results confirm that all peaks under XRD analysis matched with the International Centre for Diffraction Data (ICDD) Powder Diffraction File (PDF: 00-21-1272). The highest peak for all samples obtained at 2θ = 25.4° is the characteristic crystalline peak for pure anatase TiO_2 that follows [101] plane reflection as presented in Figure 1S. In addition, a series of crystalline peaks at 2θ = 38°, 48°, 53.8°, 55° and 62° follow [004], [200], [105], [211] and [204] planes respectively. Furthermore, no other phases (impurities) i.e., rutile and brookite, were found during the XRD analysis.
